# Evaluation of Known Markers of Ferroptosis in Semen of Patients with Different Reproductive Pathologies and Fertile Men

**DOI:** 10.3390/cells13171490

**Published:** 2024-09-05

**Authors:** Elena Moretti, Cinzia Signorini, Laura Liguori, Roberta Corsaro, Fabiola Nerucci, Marcello Fiorini, Silvia Menchiari, Giulia Collodel

**Affiliations:** 1 Department of Molecular and Developmental Medicine, University of Siena, 53100 Siena, Italy; elena.moretti@unisi.it (E.M.); laura.liguori@student.unisi.it (L.L.); roberta.corsaro@student.unisi.it (R.C.); giulia.collodel@unisi.it (G.C.); 2 Clinical Pathology Unit, Department of Cellular Therapy, Hematology and Laboratory Medicine, Azienda Ospedaliera-Senese, 53100 Siena, Italy; fabiola.nerucci@gmail.com (F.N.); marcello.fiorini@ao-siena.toscana.it (M.F.); 3 Department of Medicine, Surgery and Neurosciences, University of Siena, 53100 Siena, Italy; silvia.menchiari@gmail.com

**Keywords:** acyl coenzyme A synthetase long chain family member 4, ferroptosis, F_2_-isoprostanes, glutathione peroxidase 4, human semen, urogenital infections, varicocele

## Abstract

This study aims to investigate the role of ferroptosis, an iron-dependent form of regulated cell death, in male infertility. The motivation behind this research stems from the increasing recognition of oxidative stress and iron metabolism dysregulation as critical factors in male reproductive health. In this study, 28 infertile patients (grouped by the presence of urogenital infections or varicocele) and 19 fertile men were selected. Spermiograms were performed by light microscopy (WHO, 2021). Testosterone, ferritin, transferrin-bound iron, transferrin, and F_2_-isoprostanes (F_2_-IsoPs) were detected in seminal plasma. Glutathione peroxidase 4 (GPX4) and acyl coenzyme A synthetase long chain family member 4 (ACSL4) were also assessed in sperm cells using enzyme-linked immunosorbent assays (ELISA). All the variables were correlated (statistically significant Spearman’s rank correlations) in the whole population, and then the comparison between variables of the different groups of men were carried out. Seminal ferritin and transferrin positively correlated with seminal F_2_-IsoPs, which had positive correlations with ACSL4 detected in sperm cells. Ferritin and ACSL4 negatively correlated with the seminal parameters. No correlation was detected for GPX4. Comparing the variables in the three examined groups, elevated levels of ACSL4 were observed in infertile patients with urogenital infections and varicocele; GPX4 levels were similar in the three groups. These results suggested a mechanism of ferroptosis, identified by increased ACSL4 levels and the occurrence of lipid peroxidation. Such events appear to be GPX4-independent in reproductive pathologies such as varicocele and urogenital infections.

## 1. Introduction

Ferroptosis is an iron-dependent form of regulated cell death characterized by three fundamental actions: (1) redox activities related to iron utilization, (2) lipid peroxidation (LPO), and (3) loss or deficient repair of lipid peroxides [1,2].

In the spermatogenesis process, ferroptosis appears as a potential regulatory mechanism for maintaining the physiological number of cells at different stages within the seminiferous tubules. Overload of iron may compromise spermatogenesis as well as sperm cell organelles by inducing oxidative stress (OS) and cell death [3]. Iron plays an important role in spermatogenesis and in testosterone synthesis. Testicular iron overload can lead to the impairment of testis function responsible for spermatogenesis disorders but also to a Leydig cell dysfunction [4]. It has been suggested that the OS associated with ferroptosis could also affect the process of sperm cell development [5].

The possible role of ferroptosis in the pathophysiology of the testis may provide new insights for treating certain reproductive diseases. Lifestyle and environmental factors such as dietary iron content, oxidative stressors, and exposure to toxic substances can therefore influence the activation of ferroptosis [6]. There are pathologies of the male reproductive system connected with ferroptosis, such as testicular cancer [7], varicocele [5], cryptorchidism [8], and orchitis, in which the inflammatory responses might trigger ferroptotic processes [9].

Key players in ferroptosis include glutathione peroxidase 4 (GPX4), a critical inhibitor of LPO that reduces lipid hydroperoxides, and acyl-CoA synthetase long chain family member 4 (ACSL4), an enzyme that enhances the incorporation of polyunsaturated fatty acids (PUFAs) into phospholipids, particularly those containing arachidonic acid and adrenic acid [10,11]. GPX4 helps in maintaining an oxidative balance, and it ensures the survival of healthy germ cells and the elimination of damaged ones; ACSL4 promotes ferroptosis in germ cells. The intricate balance between GPX4 and ACSL4 is a fundamental aspect in the regulation of ferroptosis in germ cells [4]. Recently, researchers have found that inhibition of GPX4 does not entirely suppress ferroptosis in certain diseases, or cells express resistance to ferroptosis agonists that inhibit GPX4. Thus, GPX4-independent ferroptosis could be a new strategy in disease therapy [12].

Non-enzymatic oxidative damage in sperm plasma membranes, particularly those rich in PUFA, may generate prostaglandin-like end-products known as isoprostanes (IsoPs). Seminal F_2_-isoprostanes (F_2_-IsoPs), a specific class of IsoPs, have been indicated as a marker of male infertility associated with an inflammatory status and the presence of OS [13].

In this paper, the main events and molecules involved in the ferroptosis process were investigated in human seminal samples. Samples were obtained from men with different reproductive conditions, and semen parameters were assessed; then, the concentrations of GPX4 and ACSL4 were quantified in spermatozoa and the levels of molecules involved in iron metabolism, testosterone, and F_2_-IsoPs were dosed in the seminal plasma. Finally, the localization of ALOX 15, a non-heme iron-dependent dioxygenase that promotes lipid peroxidation of PUFA, was studied by immunofluorescence in ejaculated sperm.

## 2. Materials and Methods

### 2.1. Patients

In this study, our patient cohort consisted of 28 selected infertile Italian male subjects (aged 28–39), assigned into distinct groups based on their reproductive condition. These patients attended the Department of Molecular and Developmental Medicine for semen analysis. They had educational levels of high school and college, and their jobs were professional or administrative. Male infertility was defined as the inability to conceive even after 12 months of unprotected and frequent sexual intercourse. The female factor was excluded.

The inclusion criteria for this study were as follows: non-azoospermic men with normal karyotypes, without Y chromosome microdeletions and with a body mass index (BMI) of less than 25 kg/m^2^; normal serum hormonal profiles were a prerequisite. Men with any chronic diseases or those undergoing aggressive treatments such as radiotherapy and chemotherapy were not included, and neither were participants that had used antioxidant substances for at least four months before the study and those with significant tobacco use (>10 cigarettes/day) or a history of recreational drug and alcohol consumption.

The 28 selected patients were grouped as follows: 15 patients with urogenital infections, and 13 with clinically diagnosed varicocele.

Varicocele was diagnosed by a physical assessment and/or scrotal color Doppler ultrasonography. We excluded individuals with subclinical or grade 1 varicocele. Clinically asymptomatic urogenital infection was considered as present when positive bacteriological cultures were detected. Patients with both varicocele and positive semen culture were excluded from the analysis, as were patients with leukocytospermia. Patients had a left-side grade 2 or 3 varicocele, and one patient had a bilateral grade 2 varicocele. Semen samples of patients with urogenital infections were positive for *Enterococcus faecalis*, *Escherichia coli*, *Ureaplasma urealyticum*, and *Streptococcus agalactiae*.

The fertile group was composed of 19 Italian nonsmoker individuals (aged 26–40) who had fathered at least one child in the last 5 years. The fertile subjects were without chronic illness (diabetes, metabolic disorders) and not affected by infections and/or anatomical problems. All the inclusion criteria were the same as for the infertile group.

Before inclusion in this study, all subjects provided informed consent after being carefully apprised of the study objectives and procedures. This ensured ethical compliance and participant awareness of the research’s scope and implications. The study was conducted in accordance with the Declaration of Helsinki, and the protocol was approved by the Ethic Committee of Siena University Hospital (ID CEAVSE 25612).

### 2.2. Semen Analysis

Semen samples were collected by masturbation in a sterile container; samples were examined after liquefaction for 30 min at 37 °C. A part of each semen sample, recovered with sterile pipettes, was sent to the microbiological laboratory (within 1.30 h after collection) as advised by the World Health Organization (WHO) [14].

The volume, pH, sperm concentration, progressive motility (rapid and slow), normal morphology, and viability were assessed as recommended by the WHO guidelines [14]. The sperm morphology was evaluated with pre-stained Testsimplets^®^ slides (Waldeck GmbH & Co. KG, Münster, Germany). Eosin Y (CI 45380) staining was used to assess the sperm vitality; more than 300 spermatozoa per sample were examined with a light microscope, recording red-stained cells (dead) and unstained cells (vital). Leukocytospermia was identified with peroxidase staining (>1 × 10^6^ leukocytes/mL).

After the semen evaluation, samples were centrifuged at 400× *g* for 15 min to separate the seminal plasma and the spermatozoa. The aliquots were stored at −80 °C until use.

### 2.3. Biochemical Determination of Seminal Molecules Involved in Iron Metabolism

A semen aliquot of each ejaculate was centrifuged at 12,000× *g* for 5 min to obtain a sample free of cells as reported by Feng and colleagues [15], and the seminal plasma was collected by micropipette and stored in 2 mL cryotubes at −80 °C until use, then thawed at room temperature.

Seminal plasma samples (1 mL at room temperature) were tested using a COBAS 8000 modular analyzer (Roche Diagnostics GmbH, Mannheim, Germany) by means of two analytical modules: C702, the high-throughput clinical chemistry module, and E801, the immunoassay module. The following parameters were dosed in seminal plasma: iron (µg/dL), transferrin (mg/dL), ferritin (ng/mL), and testosterone (ng/mL). For the analytes measured in module C702 (iron, transferrin), COBAS 8000 calibration was performed with the human lyophilized serum calibrator C.f.a.s. (Roche Diagnostics GmbH, Mannheim, Germany). The C.f.a.s. (calibrator for automated systems) is a universal calibrator for adjusting most photometric methods. Human lyophilized serum PreciControl ClinChem level 1 was used as a normal control and PreciControl ClinChem level 2 was used as a pathological control (Roche Diagnostics GmbH, Mannheim, Germany). For the analytes measured in module E801 (ferritin, testosterone), we used specific calibrators for each analyte (Roche Diagnostics GmbH, Mannheim, Germany). PreciControl Varia levels 1 and 2 (Roche Diagnostics GmbH, Mannheim, Germany), PreciControl Universal levels 1 and 2 (Roche Diagnostics GmbH, Mannheim, Germany), and PreciControl Tumor Marker levels 1 and 2 (Roche Diagnostics GmbH, Mannheim, Germany) were used as normal and pathologic controls, respectively.

### 2.4. Acyl-CoA Synthetase Long Chain Family Member 4 (ACSL4) and Glutathione Peroxidase 4 (GPX4) Evaluation

For the ACSL4 and GPX4 evaluation, a cell lysate was prepared, adding to the semen pellet a radioimmunoprecipitation assay (RIPA) lysis buffer (300 µL, RIPA Lysis and Extraction Buffer, Thermo Fisher Scientific, Waltham, MA, USA) in the presence of protease inhibitor 1× (Halt™ Protease and Phosphatase Inhibitor Cocktail, EDTA-free Thermo Fisher Scientific, Waltham, MA, USA). The cell lysate was kept on ice for 1 h. During the cell lysate process, intermittent shaking of each sample was performed to completely lyse the cells. At the end of the cell lysate preparation, a centrifugation at 10,000× *g* for 10 min (2–8 °C) was carried out.

In the cell lysate, ACSL4 was quantified with a Human ACSL4 ELISA Kit (MyBiosource, San Diego, CA, USA, Catalog Number MBMBS2709691). Samples and a biotin-conjugated antibody specific to ACSL4 were added to the plate wells, pre-coated with an antibody specific to ACSL4. Then, avidin conjugated to horseradish peroxidase was added.

Also, GPX4 concentration was determined by ELISA (AssayGenie, Dublin, Ireland, GPX4 ELISA Kit, Human, HUFI04038) in sperm lysate. After the initial incubation, a biotinylated antibody specific for GPX4 was added, followed by the addition of an avidin–horseradish peroxidase conjugate.

In each sample, the optical densities, directly proportional to the concentrations of GPX4 and ACSL4, were measured at 450 nm and compared to the optical densities of the respective standard curves. Finally, results were reported as ng/mL for ACSL4 and GPX4. In both assays, precision and reliability were ensured, with inter-assay variability maintained below 12% and 10%, respectively. For the ACSL4 assay, sensitivity was at 0.064 ng/mL and the working range was from 0.156 to 10 ng/mL; for GPX4 detection, the test sensitivity was 46.875 pg/mL and the detection range was from 78.125 to 5000 pg/mL.

### 2.5. Seminal F_2_-Isoprostane (F_2_-IsoP) Determination

F_2_-IsoPs are initially produced as esterified to fatty acids and released into the biological fluids as free (non-esterified) F_2_-IsoPs. Here, total F_2_-IsoPs were quantified in seminal plasma with GC/NICI-MS/MS. Butylated hydroxytoluene (BHT) was added (final concentration 90 μM) to each sample at the time of freezing, and samples were stored at −80 °C until analysis. Each sample was acidified by adding 1 N HCl (1:0.5, *v*:*v*). A tetradeuterated derivative of prostaglandin (PGF_2α-d4_, 500 pg) was added as an internal standard. The sample was purified by using two different solid-phase extractions (octadecylsilane, C18 cartridge and aminopropyl, NH2 cartridge) to obtain a final eluate to be derivatized before performing GC/NICI-MS/MS. The amount of 8-iso-PGF_2α_ (also known as 15-F_2t_-IsoP), the most abundant isomer used for F_2_-IsoP evaluation, was quantified by measuring *m*/*z* 299 ion production, derived from the [M-181]-precursor ions, and compared with the *m*/*z* 303 ion produced by PGF_2α-d4_ in the applied GC/NICI-MS/MS protocol [16]. For quantification, a calibration curve was constructed using as a reference the 8-iso-PGF_2α_ compound (Item No. 16350, Caymen Chemical, Ann Arbor, MI 48108, USA). Data are reported as ng/mL.

### 2.6. Immunofluorescence Analysis

Spermatozoa were washed in phosphate-buffered saline (PBS), smeared on glass slides, and air dried. The slides were fixed in 4% paraformaldehyde in PBS for 15 min at room temperature, and treated with blocking solution (PBS–Bovine Serum Albumin (BSA) 1% + Normal Goat serum (NGS) 5%) for 20 min. Samples were then incubated with monoclonal anti-arachidonate 15-lipoxygenase (ALOX15, Life Technologies, Thermo Fisher Scientific, Eugene, OR, USA) at 1:100 in PBS-BSA 0.1% + NGS 1% at 4 °C overnight. The next day, slides were rinsed three times in PBS, and the reaction was revealed by the goat anti-mouse antibody conjugated to Alexa Fluor 568 (lot. 2124366, Life Technologies Corporation, Eugene, OR, USA) at 1:100 in PBS-BSA 0.1% + NGS 1% for 1 h at room temperature. Then, slides were washed three times with PBS for 10 min. Nuclei were stained with 4,6-diamidino-2-phenylindole (DAPI) solution (Vysis, Downers Grove, IL, USA) for 10 min. Slides were washed twice in PBS and finally mounted with 1,4-diazabicyclo-2.2.2-octane solution (DABCO, Sigma-Aldrich, Milan, Italy). Incubation in primary antibody was omitted in control samples. Slides were observed with a Leica DMI 6000 Fluorescence Microscope (Leica Microsystems, Wetzlar, Germany), and the images were acquired by the Leica AF6500 Integrated System for Imaging and Analysis. For each sample, about two hundred sperm were evaluated and the presence of the label was recorded.

### 2.7. Statistical Analysis

Statistical analysis was performed with the SPSS software package version 23.0 for Windows (SPSS Inc., Chicago, IL, USA). The Spearman’s rank correlation coefficient (rho) was used to discover the relation existing between the variables investigated. The Kolmogorov–Smirnov test was applied to verify if the variables in this study follow a normal distribution. The Levene test was performed to test homoscedasticity and to determine the appropriate post hoc test. Because some variables showed a non-normal distribution, the comparison between groups (fertile, varicocele, urogenital infections) was performed using a non-parametric test, the Kruskal–Wallis test. Then, a post hoc analysis using the Dunnett test or Tukey’s test was applied. Data are reported as the median (interquartile range [IQR]). *p* < 0.05 was considered significant.

## 3. Results

We examined the seminal samples of 47 men (19 fertile and 28 infertile due to different conditions; 15 patients with urogenital infections and 13 with varicocele). Semen characteristics, levels of seminal transferrin-bound iron, transferrin, ferritin, testosterone, and F_2_-IsoPs, and the presence of GPX4 and ACSL4 in sperm cells were assessed in all examined populations, and are reported in Table 1.

In order to understand the possible correlations among the studied variables, we used the Spearman rank correlation coefficient considering the whole population of interest (Table 2).

We found significant positive correlations between seminal transferrin (with ferritin *p* < 0.05) and transferrin-bound iron (*p* < 0.01). Ferritin and transferrin positively correlated with seminal F_2_-IsoPs (respectively *p* < 0.001 and *p* < 0.05), which had a positive correlation with ACSL4 detected in sperm cells (*p* < 0.05). Ferritin and ACSL4 showed significant negative correlations with the seminal parameters (Table 2).

Seminal levels of testosterone had a positive correlation with sperm vitality (*p* < 0.01) and a negative one with F_2_-IsoPs (*p* < 0.05, Table 2). GPX4 showed no correlations.

Semen characteristics of the infertile patients with urogenital infections or varicocele and of the fertile group are reported and compared in Table 3.

The variables iron, transferrin, and volume did not show significant differences when the three groups were compared (fertile men, men with varicocele, and men with urogenital infections).

Sperm concentration, progressive motility, normal sperm morphology, and vitality were reduced in both groups of infertile patients compared with fertile men. Moreover, the sperm vitality percentage in the urogenital infection group was significantly lower than that observed in the varicocele group.

**Figure 1:**
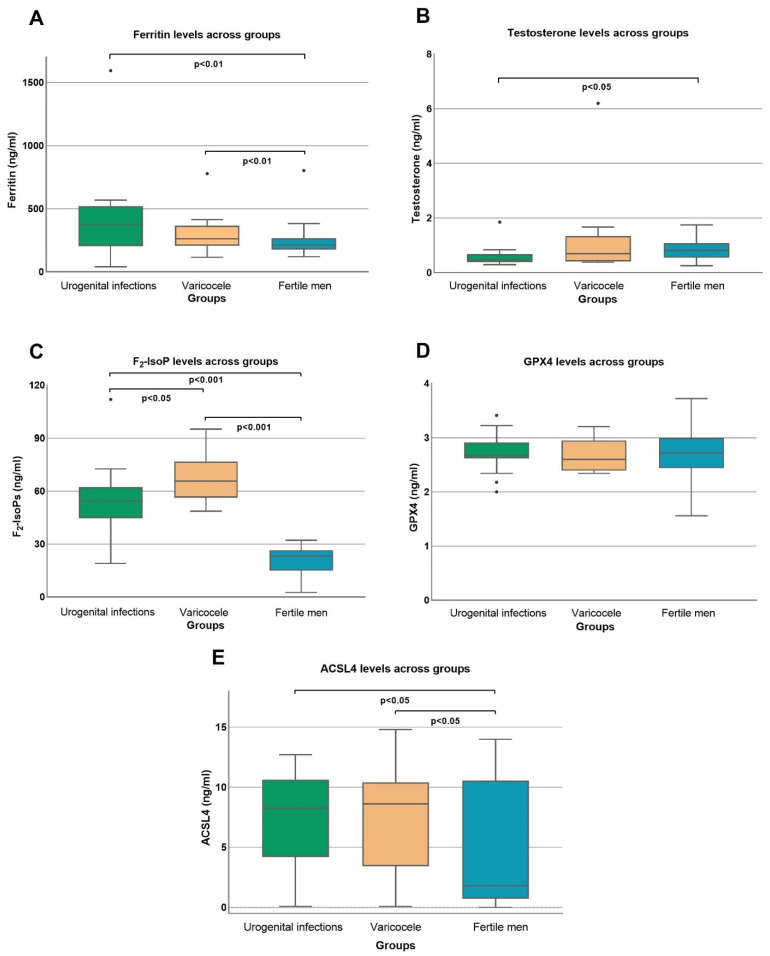
Seminal levels of ferritin (**A**), testosterone (**B**), and F_2_-isoprostanes (**C**, F_2_-IsoPs) and sperm concentrations of glutathione peroxidase 4 (GPX4, **D**) and acyl coenzyme A synthetase long chain family member 4 (ACSL4, **E**) in the three considered groups are shown.

Seminal ferritin significantly increased in both infertile groups compared with that detected in fertile men (Table 3, Figure 1A).

Interestingly, the seminal testosterone level (Figure 1B) was significantly decreased (*p* < 0.05) in the group with urogenital infections; in patients with urogenital infections or varicocele, the sperm ACSL4 concentration (Table 3, Figure 1C) was significantly increased (*p* < 0.05) compared with that observed in the fertile group. GPX4 did not show significant differences among the groups (Figure 1D).

The seminal F_2_-IsoP level, a reliable indicator of OS, was significantly higher in both infertile groups (varicocele and urogenital infection groups) than that detected in fertile men (both *p* < 0.001) and in the varicocele group compared with the urogenital infection group (Figure 1C, *p* < 0.05).

**Figure 2:**
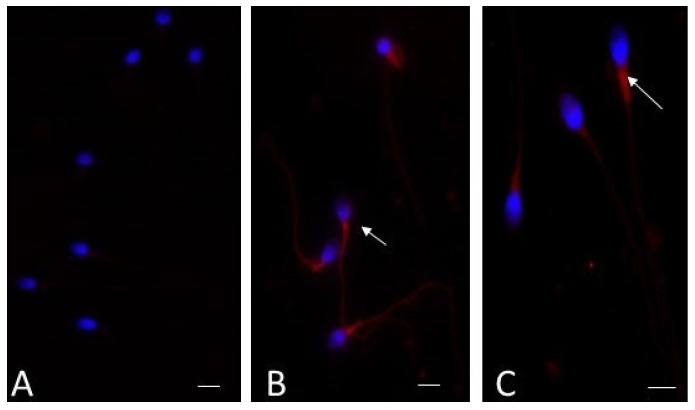
Immunocytochemical staining with ALOX 15 monoclonal antibody of sperm from a fertile man (**A**), an infertile patient with a urogenital infection (**B**), and an infertile patient with varicocele (**C**). In the sperm from a fertile man, the signal is absent; in the sperm from an infertile man with a urogenital infection, the label is evident and located in the midpiece of the sperm tail (arrow). A high percentage of spermatozoa from an infertile patient with varicocele show an intense staining in the midpiece (arrow), as do spermatozoa from a patient with an infection. Nuclei (blue) were stained with DAPI. Bar: 5 μm.

Immunofluorescence analysis showed the presence of ALOX 15 with a variable localization in spermatozoa of infertile patients and in fertile men. In spermatozoa from fertile men, the label was almost absent (95%; Figure 2A); in patients with urogenital infections, the label was present in almost 65–70% of spermatozoa and localized in the neck region and midpiece (Figure 2B). In spermatozoa from infertile patients affected by varicocele, immunofluorescence staining of ALOX 15 was present in about 55% of spermatozoa with the same localization observed in spermatozoa of patients with urogenital infections (Figure 2C).

## 4. Discussion

In this paper, the presence of ferroptosis, an iron-dependent form of regulated cell death, was assessed in normal and altered human semen. Ferroptosis caused by iron overload is closely related to OS. OS, which refers to an imbalance in levels of ROS and antioxidants, is one of the main causes of infertility in men. A small level of ROS is required for the physiological functions of sperm, such as capacitation, hyperactivation, and the acrosomal reaction. On the other hand, high levels of ROS can cause infertility through not only lipid peroxidation or DNA damage but also through inactivation of enzymes and oxidation of proteins in spermatozoa [17]. OS affecting the structural and functional integrity of sperm is a major cause of defective sperm function, of sperm death, and, consequently, of male infertility [18].

Many pathological conditions causing male infertility are associated with a high level of OS, including varicocele, obesity/metabolic syndrome, leukocytospermia, and bacterial and viral infections [18]. Many mechanisms have been associated with varicocele-related male infertility. It is believed that in varicocele an important stimulator of ROS may be a condition of hypoxia [19].

In the entire considered population, made up of fertile and infertile men, we found that seminal transferrin and ferritin positively correlated with seminal F_2_-IsoPs, a marker of OS that, in turn, had a negative correlation with sperm parameters, indicating that molecules associated with iron metabolism may alter the semen quality, inducing LPO. It is known that an increased iron uptake contributes to ROS and oxidized lipid production, and on the other hand, the negative regulators of ferroptosis reduce cellular iron uptake and LPO [20].

LPO is responsible for reduced sperm motility, alteration in the acrosome reaction, and, in some cases, damage to DNA integrity [21]. Collodel et al. [22] reported that seminal F_2_-IsoPs and the indices of iron metabolism (ferritin, iron, and transferrin) were positively associated with low sperm quality and sperm necrosis as evaluated with a transmission electron microscope (TEM).

The ultrastructural features of sperm necrosis highlighted by TEM analysis were a broken plasma membrane, disrupted chromatin, reacted acrosomes, altered axonemes, and swollen mitochondria. Sperm necrosis defined by TEM has been reported in infertile patients with inflammation [23]. Ferroptosis evaluated by TEM analysis in other cell types was characterized by the presence of shrunken mitochondria [24] or mitochondria with vacuolation, disordered and loose arrangement of cristae, and an incomplete membrane [25]. TEM analysis cannot be a completely reliable method to distinguish ferroptosis from necrosis because they share several ultrastructural characteristics. On the other hand, the assessment of specific biochemical markers is reputed to be a reliable method to identify ferroptosis. By this approach, high levels of ferroptosis, identified by glutathione (GSH), LPO, iron, and GPX4 protein, were observed in human sperm from heavy smokers [26]. These markers seemed sensitive enough for ferroptosis detection.

It is known that GPX4 is a crucial antioxidant enzyme in mammals, protecting cells from LPO whose activity requires GSH as a reducing substrate [27,28]. Recently, Hao et al. [29] suggested that a decrease in GPX4 contributed to ferroptosis in asthenozoospermic patients. In our study, GPX4 did not show any correlations, and it does not seem to indicate an involvement in the process of ferroptosis. In the literature, several GPX4-independent ferroptosis pathways are reported [12,30,31]; in certain cases, ferroptosis is not completely suppressed by inhibiting GPX4 activity.

On the other hand, in this study, ACSL4 seems to be able to identify the ferroptotic process. ACSL4 concentration, detected in sperm cells, had a positive correlation with F_2_-IsoPs. F_2_-IsoP levels, as a marker of arachidonic acid free radical-induced oxidation, increase in the presence of LPO, and this event appears to also be related to ACSL4, which is known to enrich cellular membranes with long polyunsaturated omega 6 fatty acids, such as arachidonic acid [10]. Also, ACSL4, converting fatty acids to fatty acyl-CoA esters, regulates lipid biosynthesis and contributes to ferroptosis [32].

The comparisons of investigated variables in the groups of fertile men and infertile patients with varicocele or urogenital infections confirmed the hypothesis of a pathway of GPX4-independent ferroptosis in these altered conditions. Varicocele and urogenital infections are reproductive pathologies in which an inflammatory status and OS condition are generally present [18]. As can be imagined, the infertile patients selected in this study, with either varicocele or infections, showed the worst seminal quality. Interestingly, ACSL4 was significantly higher in the groups with urogenital infections and varicocele compared to the group of fertile men; on the other hand, GPX4 concentration did not show significant differences among the groups. Moreover, the seminal F_2_-IsoP level and ferritin also increased in both infertile groups compared to fertile men; in particular, F_2_-IsoP levels were higher in the varicocele group compared with those detected in the urogenital infection group, confirming the already known association between this molecule and the condition of varicocele in infertile men [33]. These results seem to indicate that, in both infertile groups where the presence of OS was detected, GPX4-independent ferroptosis death involves the ACSL4 enzyme. Nevertheless, it is worth pointing out that ferritin levels may have more than one form of clinical significance. In fact, the amounts of ferritin are modulated by iron metabolism, so much so that ferritin levels at the plasma level are representative of iron deposits [34], but they are also indicative of acute phase response [35,36]. In fact, ferritin is an acute phase protein; thus, its increase can occur in response to an inflammatory event. Therefore, the significant increase in ferritin in the varicocele and urogenital infection groups can be linked to ferroptosis mechanisms and/or to the inflammatory response.

The presence of ferroptosis in these pathological reproductive conditions is supported by the literature [5,37,38]. Pathogens seem to regulate ferroptosis by promoting their own replication, dissemination, and pathogenesis, or evading host immune responses, although the interplay between ferroptosis and pathogenic infections is not completely clarified yet [37]. Regarding varicocele conditions, data appear discordant. The evaluation of molecular markers (Nrf2, Slc7a11, P53, and p-Jnk) indicated the absence of involvement of ferroptosis in the testes of rats with induced varicocele [38], but it has been reported that bilateral varicocele leads to ferroptosis of human spermatozoa and affects semen quality in infertile men [5].

An interesting datum in our study was the reduced level of seminal testosterone in both groups of infertile patients compared to that of fertile men, although it did not reach statistical significance in the varicocele group. In a previous paper, the indices of iron metabolism were positively associated with low sperm quality and negatively with testosterone levels in leukocytospermia and varicocele infertile patients [22]. Recently, Liang et al. [39] reported GPX4-independent ferroptosis controlled by sex hormones. In particular, they identify two phospholipid-modifying enzymes, MBOAT1 and MBOAT2, acting as ferroptosis suppressors, upregulated by sex hormone receptors. In our study, the decreased level of testosterone concomitant with increased ACSL4 concentration, and similar levels of GPX4, could suggest a GPX4-independent pathway of ferroptosis in the presence of varicocele and urogenital infection.

Finally, the immunolocalization of ALOX15, a lipoxygenase enzyme that promotes lipid degradation, confirmed the possible presence of ferroptosis in ejaculated sperm of infertile patients with varicocele and urogenital infections. In infertile patients, the strong signal of ALOX15 in the midpiece of the sperm tail seems to indicate mitochondrial involvement. The morphological characteristics of ferroptosis include changes in the mitochondrial membrane and cristae loss. Mitochondria are responsible for energy and ROS production in eukaryotic cells; they are involved in supporting spermatogenesis, sperm motility, and apoptosis regulation [40]. ALOX15 is the key enzyme in the formation of 4-Hydroxynonenal, an important product of plasma membrane LPO, which is a cause of cell and tissue injury [41]. Sperm from fertile men did not show the presence of ALOX 15, which was clearly expressed in sperm from infertile men with either varicocele or urogenital infections, where the increased levels of F_2_-IsoPs confirmed the presence of OS and LPO. ALOX15 and ACSL4 were shown to be upregulated in response to OS in mouse round spermatids in an in vitro study to test differential cell death in the testis [42].

The results of this research seem to confirm the presence of ferroptosis in sperm from patients with urogenital infections and varicocele, also indicating LPO damage due to the condition of OS.

The involvement of ferroptosis pathways could suggest that the use of antioxidants or iron chelators, inhibiting the ferroptosis process, may prevent and treat testicular diseases, improving male reproductive function. This idea has been tested in various animal models; in humans, few clinical trials have been conducted to evaluate the therapeutic effects of ferroptotic inhibitors on male reproductive diseases [4].

We are aware that this study involves a small number of patients, which will need to be increased; however, the strict selection criteria make the groups reliable.

## 5. Conclusions

In conclusion, it is fundamental to underline the relevance of studying the process of ferroptosis, which is still only partially known in male infertility.

We suggest a role of the mechanisms of ferroptosis in sperm of infertile patients with urogenital infections and varicocele; further studies need to clarify if ferroptosis can co-exist with other types of cell death. This will allow identification of specific markers of ferroptosis for the development of possible treatments and improve our knowledge of the role of mechanisms of ferroptosis in reproductive male pathological conditions.

## Figures and Tables

**Table 1:** Medians and 25–75° centiles (in parentheses) of all the variables assayed in the seminal fluid or sperm of 47 men.

Variables	Median
**Semen parameters**	
Volume (mL)	3.00 (2.20–4.00)
Sperm/mL ×10^6^	46.00 (19.00–62.00)
Sperm progressive motility %	25.00 (17.00–43.00)
Sperm normal morphology %	8.00 (5.00–14.00)
Sperm vitality %	73.00 (62.00–82.00)
**Seminal plasma**	
Ferritin (ng/mL)	241.00 (184.00–413.00)
Iron (µg/dL)	16.20 (13.00–24.40)
Trasferrin (mg/dL)	1.00 (0.00–4.00)
Testosterone (ng/mL)	0.69 (0.46–0.91)
F_2_-isoprostanes (F_2_-IsoPs, ng/mL)	46.43 (25.12–61.90)
**Spermatozoa**	
Glutathione peroxidase 4 (GPX4, ng/mL)	2.7 (2.47–2.94)
Acyl coenzyme A synthetase long chain family member 4 (ACSL4, ng/mL)	6.41 (1.30–10.35)

**Table 2:** Correlations (rho Sperman’s coefficient) between all considered variables in 47 individuals. Statistics are also reported: * *p* < 0.05, ** *p* < 0.01, *** *p* < 0.001.

	Seminal Ferritin (ng/mL)	Seminal Iron (µg/dL)	Seminal Trasferrin (mg/dL)	Seminal Testosterone (ng/mL)	Sperm Glutathione Peroxidase 4 (GPX4, ng/mL)	Sperm Acyl Coenzyme A Synthetase Long Chain Family Member 4 (ACSL4, ng/mL)	Volume (mL)	Sperm/ mL ×10^6^	Sperm Progressive Motility %	Sperm Normal Morphology %	Sperm Vitality %	Seminal F_2_-Isoprostanes (ng/mL)
Seminal ferritin (ng/mL)	1											
Seminal iron (µg/dL)	0.175	1										
Seminal trasferrin (mg/dL)	0.351 *	0.427 **	1									
Seminal testosterone (ng/mL)	−0.278	−0.101	−0.180	1								
Sperm glutathione peroxidase 4 (GPX4, ng/mL)	0.163	−0.113	0.063	−0.228	1							
Sperm acyl coenzyme A synthetase long chain family member 4 (ACSL4, ng/mL)	0.263	−0.183	−0.087	−0.103	−0.114	1						
Volume (mL)	−0.175	−0.127	−0.131	−0.133	0.195	−0.224	1					
Sperm /mL ×10^6^	−0.222	0.139	0.028	0.337 *	0.221	−0.112	0.131	1				
Sperm progressive motility %	−0.685 ***	−0.144	−0.097	0.179	0.207	−0.385 **	0.237	0.486 ***	1			
Sperm normal morphology %	−0.649 ***	−0.042	−0.165	0.280	0.219	−0.570 ***	0.232	0.613 ***	0.743 ***	1		
Sperm vitality %	−0.481 ***	−0.207	−0.252	0.364 **	0.267	−0.468 ***	0.279	0.471 ***	0.605 ***	0.621 ***	1	
Seminal F_2_-isoprostanes (ng/mL)	0.780 ***	0.198	0.306*	−0.288 *	−0.201	0.286 *	−0.146	0.167	−0.850 ***	−0.736 ***	−0.501 ***	1

**Table 3:** Medians and interquartile ranges (in parentheses) of semen parameters, seminal ferritin, transferrin-bound iron, transferrin, testosterone, F_2_-isoprostanes (F_2_-IsoPs), and sperm concentrations of acyl coenzyme A synthetase long chain family member 4 (ACSL4) and glutathione peroxidase 4 (GPX4) assayed in semen samples of 47 men divided into 3 groups according to their clinical condition (urogenital infections, 15 men; varicocele, 13 men; fertile, 19 men). Statistics are also reported.

	Urogenital Infections (UI, n. 15)	Varicocele (V, n. 13)	Fertile (F, n. 19)	Statistics	
				Kruskal–Wallis	Tukey or Dunnet Test
Volume (mL)	3.00 (2.20–4.00)	3.90 (2.00–4.00)	3.50 (2.50–4.40)	NS	
Sperm/mL ×10^6^	18.00 (6.00–25.00)	32.00 (14.00–55.00)	56.00 (48.00–68.00)	*p* < 0.001	F vs. UI (*p* < 0.01) F vs. V (*p* < 0.05)
Sperm progressive motility %	21.00 (12.00–26.00)	17.00 (13.50–18.50)	43.00 (40.00–50.00)	*p* < 0.001	F vs. UI (*p* < 0.001) F vs. V (*p* < 0.001)
Sperm normal morphology %	6.00 (3.00–7.00)	6.00 (4.00–8.00)	14.00 (13.00–18.00)	*p* < 0.001	F vs. UI (*p* < 0.001) F vs. V (*p* < 0.001)
Sperm vitality %	60.00 (55.00–71.00)	70.00 (64.00–79.00)	81.00 (75.00–90.00)	*p* < 0.001	F vs. UI (*p* < 0.001) F vs. V (*p* < 0.050) UI vs. V (*p* < 0.050)
**Seminal determinations**					
Ferritin (ng/mL)	432.0 (341.0–533.0)	344.0 (235.0–414.0)	183.0 (167.9–210.0)	*p* < 0.001	F vs. UI (*p* < 0.01) F vs. V (*p* < 0.01)
Iron (µg/dL)	17.30 (13.70–28.40)	16.00 (10.40–24.15)	16.20 (13.00–24.00)	NS	
Transferrin (mg/dL)	1.00 (0.00–6.00)	2.00 (0.00–4.50)	0.00 (0.00–2.00)	NS	
Testosterone (ng/mL)	0.463 (0.389–0.644)	0.693 (0.409–1.345)	0.855 (0.665–1.140)	*p* < 0.01	F vs. UI (*p* < 0.05)
F_2_-IsoPs (ng/mL)	53.23 (45.24–61.90)	65.78 (56.29–76.79)	23.56 (14.07–26.65)	*p* < 0.001	F vs. UI *p* < 0.001) F vs. V (*p* < 0.001) V vs. UI (*p* < 0.05)
**Spermatozoa determinations**					
ACSL4 (ng/mL)	8.25 (4.925–10.425)	8.6 (4.62–10.2)	1.8 (0.775–10.05)	*p* < 0.001	F vs. UI (*p* < 0.05) F vs. V (*p* < 0.05)
GPX4 (ng/mL)	2.68 (2.62–2.89)	2.60 (2.40–2.80)	2.72 (2.47–2.99)	NS	

## Data Availability

The data generated and analyzed during this study are included in this published article and are available from the corresponding author.
